# The Effectiveness of a Stoicism‐Based Educational Package on Psychological Distress and Self‐Efficacy in Patients Under Hemodialysis: A Randomized Controlled Trial

**DOI:** 10.1002/hsr2.73336

**Published:** 2026-10-05

**Authors:** Amirhossein Boostani, Amirmohamad Momeni, Mohammad‐Ali Cheraghi, Fatemeh Salmani, Mozhgan Moshtagh

**Affiliations:** ^1^ Instructor of Nursing, Department of Nursing, Ferdows Faculty of Medical Sciences, Chamran Hospital of Ferdows Birjand University of Medical Sciences Birjand Iran; ^2^ Student Research Committee Birjand University of Medical Sciences Birjand Iran; ^3^ Department of Critical Care and Nursing Management, Spiritual Health Group Tehran University of Medical Sciences Tehran Iran; ^4^ Department of Epidemiology and Biostatistics, Faculty of Health, Geriatric Health Research Center Birjand University of Medical Sciences Birjand Iran; ^5^ Social Determinants of Health Research Center Birjand University of Medical Sciences Birjand Iran

**Keywords:** hemodialysis, psychological distress, self‐efficacy, stoicism

## Abstract

**Background and Aims:**

Patients undergoing hemodialysis face numerous challenges and adversities, which can impact their mental health. Several previous studies have indicated that certain Stoic tenets may be beneficial in alleviating suffering and enhancing mental health outcomes in situations such as chronic disease. Therefore, this study explored the effectiveness of Stoic training in reducing psychological distress and enhancing self‐efficacy among hemodialysis patients.

**Methods:**

This randomized controlled trial was conducted from February to May 2025. The 8‐week intervention began with virtual Stoic training sessions and transitioned to weekly in‐person meetings. Data were collected using a demographic questionnaire, the Kessler Psychological Distress Questionnaire (K‐10), and the Self‐Efficacy for Managing Chronic Diseases 6‐Item Scale. Data were analyzed using SPSS version 22. Independent *t*‐tests, *χ* ^2^ tests, and multiple linear regression were used for analysis.

**Results:**

A total of 50 participants (25 in each group) were analyzed. The multiple linear regression model, controlling for baseline scores, hypertension, and education level, showed that the intervention was significantly associated with a reduction in psychological distress (*β* = 4.75, *p *= 0.02, 95% CI [8.88, 0.61]) and an increase in self‐efficacy for managing chronic disease (*β* = 8.76, *p *= 0.009, 95% CI [2.51, 15.01]).

**Conclusion:**

The findings suggest that while Stoic training can help alleviate psychological distress, further research is needed to develop an expanded training protocol. This may involve longer practice durations and more scenario‐based exercises to significantly enhance self‐efficacy. Researchers should consider these factors in future studies.

**Abbreviations**
PD: psychological distress
SE: self‐efficacy for managing chronic disease

## 1 Introduction

Chronic kidney disease poses a significant global public health challenge, impacting over 697 million people worldwide by 2019. Millions of new cases are reported each year, frequently progressing to end‐stage renal disease (ESRD), which necessitates treatments like hemodialysis [1]. In Iran, the incidence of ESRD has risen by approximately 11% annually, with an estimated prevalence of 250 cases per million population [2]. Patients undergoing hemodialysis often encounter a range of physical, psychological, social, and financial problems that can lead to psychological distress. Therefore, enhancing the quality of life for these patients is crucial [3, 4].

Psychological distress, characterized by emotions such as depression, anxiety, and anger, arises when individuals feel unable to manage frustrating or threatening circumstances [5]. This disorder is highly prevalent among hemodialysis patients and is associated with poorer quality of life and increased mortality [6, 7]. A 2017 systematic review estimated the prevalence of depression in Iranian hemodialysis patients at 62% [8]. Evidence suggests that lower levels of depression and psychological distress in hemodialysis patients predict better adherence to treatment [9] and significantly contribute to an improved quality of life. Consequently, implementing interventions to manage psychological distress is vital in this patient population.

Self‐efficacy refers to a person's belief in their capacity for managing diverse situations, including self‐care, dietary management, and treatment adherence [10]. Higher self‐efficacy in hemodialysis patients has been linked to better self‐care, improved treatment adherence, and enhanced weight management between dialysis sessions. These benefits can lead to fewer hospitalizations, reduced anxiety and depression, and a better quality of life [11]. Unfortunately, hemodialysis patients often report lower perceived self‐efficacy regarding their condition compared to those on peritoneal dialysis [12]. Therefore, developing strategies to boost self‐efficacy in hemodialysis patients could significantly improve their physical and mental well‐being.

In recent years, there has been growing interest in non‐pharmacological psychological interventions, such as those rooted in philosophy and meaning‐centered education. Applied philosophy aims to alter distressing beliefs and behaviors using related theories and practices [13]. For example, Stoicism, the ancient Greek philosophy, emphasizes inner peace by focusing on personal responsibility and rational responses to challenges, while accepting what is beyond individual control [14]. These principles can be especially beneficial when facing uncertainties about health and treatment outcomes, as well as the broader difficulties of living with an illness.

Some studies have shown that Stoic philosophy can improve psychological indicators such as resilience, self‐efficacy, psychological well‐being, empathy, and spiritual coping strategies in various populations, including dialysis patients [15, 16, 17].

Mindfulness, a core Stoic teaching, has shown promise in reducing stress and anxiety among hemodialysis patients, as evidenced by a 2021 study during the coronavirus outbreak [18, 19]. Furthermore, MacLellan et al. demonstrated that Stoic training can decrease rumination in anxious individuals and boost self‐efficacy [20]. Existing evidence suggests that strategies like re‐evaluating negative thoughts and concentrating on controllable aspects of one's experience can enhance patients' confidence in managing symptoms and maintaining daily activities [20]. This aligns with self‐efficacy theory, which posits that individuals who believe in their ability to achieve a positive outcome are more likely to manage their illness [12]. Implementing stress management techniques and developing adaptive coping strategies can improve the self‐efficacy of hemodialysis patients [11]. Stoic teachings represent a potential stress management intervention that requires further exploration, particularly in countries like Iran, which has a distinct sociocultural context compared to Western societies that pioneered Stoic philosophy, such as Greece [21]. Regarding the effects of Stoicism on psychological outcomes, while historical evidence‐based narrative and review studies exist, there appears to be insufficient empirical intervention research. Thus, further experimental intervention studies are recommended across diverse settings, examining different psychological indicators in varied populations such as caregivers, healthcare workers, chronic patients, and students. Given the significant psychological burden faced by hemodialysis patients and the importance of non‐pharmacological interventions, this study aimed to examine the effect of Stoic teachings on psychological distress and self‐efficacy in this patient population.

## 2 Materials and Methods

### 2.1 Design

This randomized controlled clinical trial was conducted among hemodialysis patients at the Special Diseases Center in Birjand city from February to May 2025. The primary objective of this trial was to compare changes in psychological distress and self‐efficacy from baseline to post‐intervention between the Stoicism‐based intervention group and the control group. The secondary objective was to examine the intervention's effect while controlling for potential confounders.

### 2.2 Participants

We calculated the sample size for interventional studies using the formula based on statistical parameters from Moodi et al. [22] (*α* = 0.05, *β* = 0.2, S1 = 2.45, S2 = 1.88, M1–M2 1.8) for a two‐sided independent *t*‐test. This calculation indicated that 24 subjects were needed per group. To account for a 10% dropout rate, we increased the number to 27 subjects per group.

The parameters from Moodi et al. [22] were considered suitable due to the similarity in the study population (hemodialysis patients) and the conceptual overlap between Stoic teachings and spiritual therapy.

The study commenced upon receiving a code of ethics from the Ethics Committee of Birjand University of Medical Sciences (IR.BUMS.REC.1403.275). This trial was approved with the identifier IRCT20241229064210N4 on the Iranian Registry of Clinical Trials website on March 25, 2025.

All participants signed written informed consent forms to participate in the study. They were informed that their participation was voluntary and assured that any information they provided would be kept confidential.

### 2.3 Sampling

We used a convenience sampling method to select participants, then randomly assigned them to either the intervention or control group in a 1:1 allocation ratio using random permuted blocks with quadruple blocks (creating six permutations for groups A and B to ensure equal sample sizes). The allocation sequence was concealed using opaque, sealed envelopes. A researcher (AHB) handled the registration and allocation process to ensure randomization. Participants were informed of their group assignment via telephone. Another researcher (AMM) who was assessing outcomes was blinded to assignment to interventions.

To be included in the study, participants needed to meet the following criteria:

– Provide informed consent.
– Be between 18 and 65 years of age.
– Have no history of drug addiction.
– Take no psychotic drugs or have no acute psychotic disorder.
– Have been undergoing dialysis for a minimum of 3 months.
– Be Persian‐speaking.

Exclusion criteria also included missing more than two training sessions, withdrawing from the study, worsening the disease to the extent that it prevented continued participation, and the death of the participant.

### 2.4 Materials

Psychological distress and self‐efficacy in patients on hemodialysis were the main outcomes of the study.

The demographic questionnaire collected general information about participants, including age, gender, marital status, education level, and income. It also gathered specific details related to their hemodialysis treatment, such as duration of hemodialysis, type of vascular access, number of hemodialysis sessions per week, average duration of hemodialysis per session, and their employment status. Information on comorbidities and medications was also recorded from medical charts (diabetes, hypertension, hyperlipidemia, and heart disease were considered comorbidities). Assessing psychological distress, we used the Kessler Psychological Distress Questionnaire (K‐10), developed in 2002. This instrument features 10 questions, each rated on a 5‐point Likert scale ranging from never (0 points) to always (4 points). The total score can range from 0 to 40. The Persian version of the K‐10 has demonstrated strong validity and reliability, with Cronbach's alpha values of 0.93 in Yaghubi's study [23] and 0.92 in Hajebi et al.'s study [24]. It has also been successfully applied in studies involving hemodialysis patients [25].

The Self‐Efficacy for Managing Chronic Diseases 6‐Item Scale is rated on a 10‐point Likert scale, where 1 signifies not at all confident and 10 signifies confident. The final score for this questionnaire is calculated by averaging the responses to all questions. The questionnaire has shown robust reliability in Iran, with a Cronbach's alpha coefficient of 0.9. Factor analysis further confirmed its single‐factor structure, explaining 69.49% of the extracted variance [10].

### 2.5 Procedure

This study was structured into three distinct phases: pre‐intervention, intervention, and post‐test. Initially, participants from both the control and intervention groups joined separate online groups via social networks. In this phase, all participants completed the baseline questionnaires. Before the intervention began, a qualified researcher, supervised by consulting professors, compiled the educational content. This content was based on reliable Stoic texts such as Love of Destiny, The Stoic Art of Living, The Discourses of Epictetus, and Meditations of Marcus Aurelius, along with insights from Connery et al.'s study [19]. The compiled material was then converted into a podcast series.

The intervention involved introducing participants to Stoic teachings. The first session was held in person, allowing participants to get acquainted and learn about the study's goals and session structure. Over the next 2 months, eight educational sessions (virtual and in‐person) were conducted. A weekly 20‐min podcast was uploaded to a private virtual group created for the intervention participants on a social networking platform, and they were encouraged to listen. This was followed by an individual in‐person session at the end of each week, led by the qualified researcher. These sessions were dedicated to reviewing the podcast content, fostering discussions, answering questions, and providing practical scenarios for participants to practice. The final week concluded with a summary session. Adherence to the virtual component was monitored through confirmation of podcast listening, while attendance at in‐person sessions was recorded. The research team would make telephone calls to the members of the intervention group to check their status regarding participation or non‐participation in in‐person sessions and listening to the podcasts. In the application used, it was possible to determine how many podcasts each participant had played and listened to. Once 90% of the group members had listened to a podcast, the second podcast was uploaded, and the remaining 10% were followed up by telephone to encourage them to listen to the podcast as soon as possible.

One month after the intervention concluded, both the intervention and control groups re‐completed the research questionnaires. In order to prevent information leakage to the control group during the study, face‐to‐face sessions for the intervention group were held at times when the control group members were not present at the treatment center. To adhere to ethical considerations, the control group was also given access to the Stoic teaching podcasts after the study. The detailed outline and content of the Stoic teaching intervention are presented in the Supporting Information S1 (Teachings outline). Patients undergoing hemodialysis often experience depression, fatigue, or lassitude. Consequently, their participation in surveys can be challenging. Therefore, only a post‐intervention assessment was conducted 1 month after the intervention [26, 27, 28]. A longer post‐intervention follow‐up was not conducted due to time and resource limitations inherent in the study framework. This trial was reported in accordance with the CONSORT 2010 Statement.

### 2.6 Analysis Plan

In this trial, the primary outcomes were post‐training scores for psychological distress and Self‐Efficacy for Managing Chronic Disease.

Statistical analyses were primarily based on all participants with observed post‐intervention outcome data (available‐case analysis). Descriptive statistics and between‐group comparisons reported in the tables correspond to this available‐case population.

To assess the robustness of the findings to missing data and to approximate an intention‐to‐treat (ITT) approach, we additionally conducted analyses including all randomized participants. Missing post‐intervention outcome data were handled using multiple imputation. Five imputed datasets were generated, and results were pooled to obtain overall intervention effects and associated *p* values.

All randomized participants were included in the ITT analysis and were retained in their originally assigned groups regardless of adherence or withdrawal. The primary efficacy analysis presented in the manuscript is based on all participants with observed data (available‐case analysis). ITT analyses based on the pooled multiply imputed datasets are reported alongside the available‐case results, with both ITT (pooled) and available‐case *p* values provided in the tables. The normality of the data was assessed using the Kolmogorov–Smirnov test. Quantitative variables were reported as means and standard deviations, while qualitative variables were presented as counts and percentages. An independent *t*‐test, Mann–Whitney U, *χ* ^2^ test, and Fisher's Exact test were used to compare the two groups at baseline. Paired sample *t*‐tests were conducted to compare within‐group changes from pre‐ to post‐test. The analysis of between‐group differences was conducted using multiple linear regression, with the post‐intervention score as the outcome, treatment‐group assignment as the predictor, and baseline score, hypertension status, and education level as covariates. All statistical tests were two‐sided, with a priori significance level set at *p *< 0.05. Data were analyzed using SPSS version 22.

## 3 Results

A total of 54 patients undergoing hemodialysis were recruited for this study and randomly assigned to either the intervention group (*N *= 27) or the control group (*N *= 27). Both intervention and control groups experienced dropouts, with two patients from each group (Figure 1). No adverse events were reported during the trial.

**Figure 1:**
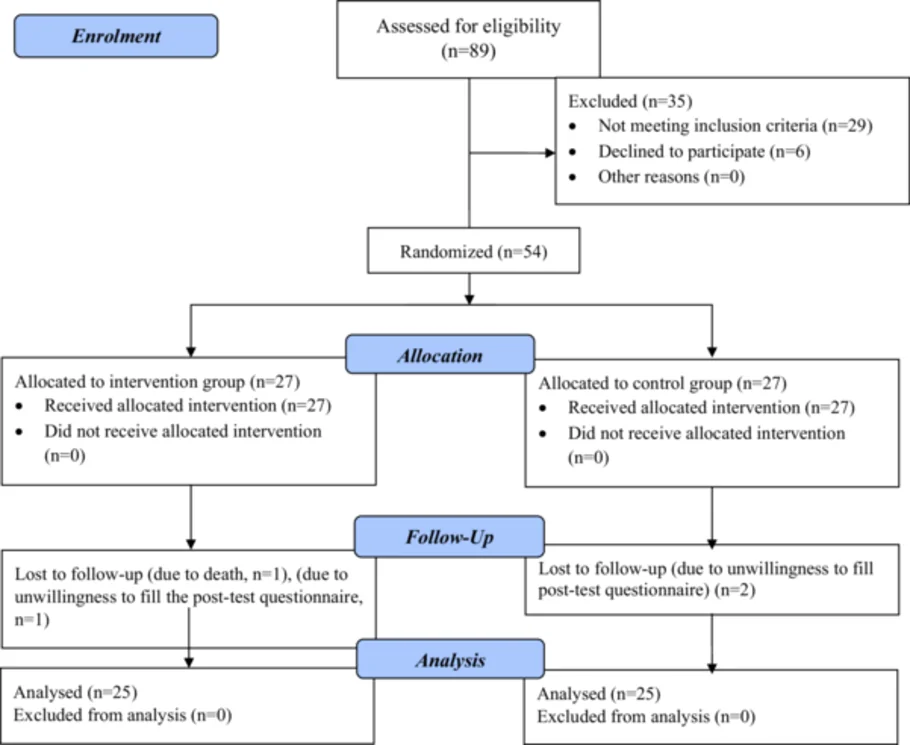
CONSORT diagram.

As indicated in Table 1, the socio‐demographic and baseline clinical characteristics were the same for both groups (*p *> 0.05).

**Table 1:** Comparison of demographic characteristics between patients undergoing hemodialysis in the two groups.

Variables		Intervention group		Control group		*Z* value	*p* value^a^
		M±SD	*m* (IQR)	M±SD	*m* (IQR)		
Age (years)		45.32 ± 14.45	43 (28)	54.04 ± 13.72	62 (25.5)	−1.92	0.54
Disease duration (years)		3.93 ± 2.90	3 (4.9)	2.73 ± 2.20	2 (3.5)	1.66	0.09
Number of hemodialysis per week		2.84 ± 0.37	3 (0)	2.68 ± 0.55	3 (1)	1.06	0.28
Time of one hemodialysis (hours)		3.70 ± 0.40	4 (0.5)	3.50 ± 0.52	3.5 (1)	1.42	0.15
Comorbidity (number)		1.04 ± 1.30	0 (2)	1.72 ± 1.36	2 (2.5)	−1.73	0.08

	Content	*n* (%)		*n* (%)		*χ* ^2^ value	*p* value^b^
Gender	Female	9 (36)		14 (56)		2.01	0.15
	Male	16 (64)		11 (44)			
Marital status	Single	6 (24)		6 (24)		0.00	1.00
	Married	19 (76)		19 (76)			
Educational level	Elementary	2 (8)		7 (28)		8.46	**0.03**
	High school	4 (16)		9 (36)			
	Diploma and associate degree	12 (48)		7 (28)			
	Bachelor's degree and above	7 (28)		2 (8)			
Place of residence	Urban	20 (80)		22 (88)		—	0.70
	Rural	5 (20)		3 (12)			
Income level	Inadequate	12 (48)		13 (52)		0.08	0.77
	Moderate	13 (52)		12 (48)			
	Adequate	0 (0)		0 (0)			
Occupation status	Employed	5 (20)		2 (8)		3.07	0.23
	Unemployed	11 (44)		17 (68)			
	Retired	9 (36)		6 (24)			
Vessel access type for hemodialysis	Fistula	19 (76)		13 (52)		3.12	0.07
	Shaldon	6 (24)		12 (48)			
Comorbidity	Yes	12 (48)		18 (72)		3.00	0.08
	No	13 (52)		7 (28)			
Diabetes	Yes	10 (40)		14 (56)		1.28	0.25
	No	15 (60)		11 (44)			
Hyperlipidemia	Yes	3 (12)		7 (28)		2.00	0.15
	No	22 (88)		18 (72)			
Heart disease	Yes	2 (8)		3 (12)		—	1.00
	No	23 (92)		22 (88)			
Hypertension	Yes	6 (24)		13 (52)		4.16	**0.04**
	No	19 (76)		12 (48)			
Using medications besides hemodialysis drug	Yes	12 (48)		18 (72)		3.00	0.08
	No	13 (52)		7 (28)			

*Note: p* < 0.05, the difference was statistically significant. Bold *p* values correspond to *p* < 0.05.

^a^ Mann–Whitney U test.

^b^ *χ* ^2^ test or Fisher's exact test where appropriate.

Post‐intervention scores for psychological distress (PD) and self‐efficacy (SE) were significantly higher in the intervention group (10.20 ± 8.88 and 45.00 ± 10.02, respectively) than in the control group (15.16 ± 6.92 and 34.16 ± 9.34, respectively; *p *< 0.05, Table 2).

**Table 2:** Comparison of changes in study outcomes before/after intervention.

Content	Pre‐test							Post‐test								
	Intervention group (*n* = 25)		Control group (*n* = 25)		*t* value	*p* value^a^	Cohen's *d*	Intervention group (*n* = 25)			Control group (*n* = 25)		*t* or Z value	*p* value	*p* value*	Cohen's *d*
	M±SD	*m* (IQR)	M±SD	*m* (IQR)				M±SD		*m* (IQR)	M±SD	*m* (IQR)				
Psychological distress	15.24 ± 10.19	13 (16.5)	13.72 ± 6.54	14 (10)	−0.62	0.53	−0.17	10.20 ± 8.88		7 (12)	15.16 ± 6.92	15 (10.5)	−2.50	**0.01** b	0.012	−0.35
Self‐efficacy	36.08 ± 12.30	36 (18.5)	31.44 ± 9.46	30 (13)	−1.49	0.14	−0.42	45 ± 10.02		47 (15)	34.16 ± 9.34	37 (17.5)	−3.95	**< 0.001** a	< 0.001	−1.11

*Note:* Cohen's *d* (Effect size) is interpreted as follows: 0–0.2 (negligible), 0.2–0.5 (small), 0.5–0.8 (moderate), and >0.8 (large).

*p* < 0.05, the difference was statistically significant. Bold *p* values correspond to *p* < 0.05.

^a^ Independent‐samples *t*‐test.

^b^ Mann–Whitney U test.

* Intention to treatment *p* value.

Table 3 presents the within‐group mean changes. While the control group showed no significant changes in either PD or SE scores (*p *> 0.05), the intervention group demonstrated a significant decrease in PD scores (pre: 15.24 ± 10.19; post: 10.20 ± 8.88; *p *< 0.05) and a significant increase in SE scores (pre: 36.08 ± 12.30; post: 45.00 ± 10.02; *p *< 0.05).

**Table 3:** Comparison of mean changes in study outcomes within groups at endline.

Content	Intervention group (*n* = 25)								Control group (*n* = 25)						
	Pre‐test		Post‐test		*p* value^a^	*p* value*	Cohen's *d*	Pre‐test			Post‐test		*p* value^a^	*p* value*	Cohen's *d*
	M±SD	*m* (IQR)	M±SD	*m* (IQR)				M±SD		*m* (IQR)	M±SD	*m* (IQR)			
Psychological distress	15.24 ± 10.19	13 (16.5)	10.20 ± 8.88	7 (12)	**0.003**	**< 0.001**	−0.65	13.72 ± 6.54		14 (10)	15.16 ± 6.92	15 (10.5)	0.34	0.195	0.19
Self‐efficacy	36.08 ± 12.30	36 (18.5)	45 ± 10.02	47 (15)	**0.007**	**< 0.001**	0.59	31.44 ± 9.46		30 (13)	34.16 ± 9.34	37 (17.5)	0.13	0.075	0.30

*Note:* Cohen's *d* (effect size) is interpreted as follows: 0–0.2 (negligible), 0.2–0.5 (small), 0.5–0.8 (moderate), and >0.8 (large).

*p* < 0.05, the difference was statistically significant. Bold *p* values correspond to *p* < 0.05.

^a^ Paired‐samples *t*‐test.

* Intention‐to‐treat *p* value.

Table 4 shows that the homogeneity of hypertension and educational levels across the groups was assessed using the chi‐square test (*p* = 0.04) and Fisher's Exact test (*p* = 0.03). Multiple regression analysis showed that, after controlling for pretest scores, hypertension, and education level, the intervention was significantly associated with a reduction in psychological distress (*β* = 4.7, *p* = 0.02) and an increase in self‐efficacy for managing chronic disease (*β* = 8.7, *p* = 0.009).

**Table 4:** Multiple linear regression models for psychological distress and self‐efficacy.

Content	Predictor variables		Coefficients	CI 95%	Standardized coefficients	*t* value	*p* value
Psychological distress^a^	PD pre‐test score		0.58	(0.36, 0.79)	0.6	5.18	**<0.001**
	Group (baseline = control)		−4.75	(−8.88, −0.61)	−0.29	−2.25	**0.02**
	Hypertension (baseline = no)		3.51	(−0.56, 7.58)	0.2	1.68	0.09
		Education level— elementary	−1.28	(−7.68, 5.13)	−0.06	−0.39	0.69
	Education (baseline = bachelor's degree and above)	Education level—high school	−0.83	(−6.78, 5.12)	−0.04	−0.27	0.78
		Education level— diploma and associate degree	−2.66	(−8.09, 2.77)	−0.15	−0.96	0.34
Self‐efficacy^b^	SE pre‐test score		0.27	(0.03, 0.51)	0.27	2.16	**0.03**
	Group (baseline = control)		8.76	(2.51, 15.01)	0.4	2.74	**0.009**
	Hypertension (baseline = no)		−0.02	(−6.02,5.97)	−0.001	−0.01	0.99
		Education level— elementary	−5.42	(14.9, 4.07)	−0.19	−1.11	0.27
	Education (baseline = bachelor's degree and above)	Education level—high school	−3.39	(−12.21, 5.43)	−0.13	−0.75	0.45
		Education level— diploma and associate degree	−4.82	(−12.83, 3.19)	−0.21	−1.18	0.24

*Note:* Bold *P*‐values correspond to *R* < 0.05.

a ADJ.R2 = 0.387, R2 = 0.462, *R* = 0.680.

b ADJ.R2 = 0.248, R2 = 0.340, *R* = 0.583.

## 4 Discussion

This study examined the influence of a Stoicism‐Based Educational Package on psychological distress and self‐efficacy in hemodialysis patients. The findings revealed a significant reduction in psychological distress, as evidenced by the *t*‐test analysis of mean change scores (*p *= 0.004, Cohen's *d* = 0.85), which was the pre‐specified primary analysis in the trial registration.

Regarding self‐efficacy, the pre‐specified *t*‐test analysis of mean change scores did not show a statistically significant improvement (*p *= 0.08, Cohen's *d* = −0.50). However, a secondary multiple linear regression analysis, which adjusted for baseline scores and confounding variables (hypertension and education level), revealed a significant positive effect of the intervention on self‐efficacy (*β* = 8.76, *p *= 0.009, 95% CI [2.51, 15.01]). This discrepancy between the primary and secondary analyses may be attributed to the greater statistical power and precision of the regression model in controlling for baseline imbalances and covariates. Given that the primary outcome was pre‐specified as the *t*‐test of change scores in the trial registration, we have prioritized this finding in our interpretation. Nonetheless, the secondary regression results suggest that Stoic training may have a beneficial effect on self‐efficacy when potential confounders are accounted for, warranting further investigation in future studies with larger sample sizes and extended follow‐up periods.

Given the limited body of research on Stoicism, comparative analyses were drawn from studies most closely aligned with the present investigation.

Key strengths of the current study include an adequate sample size, a randomized control group, and follow‐up assessments—features that distinguish it from prior research.

In the present study, psychological distress demonstrated significant improvement. This finding aligns with the longitudinal study by Akrim et al., which demonstrated that Stoic practices reduced psychological distress among healthcare system employees [29]. Similarly, Hajizadeh et al. found that Stoic training enhanced resilience among operating room staff [30].

Brown et al. demonstrated that Stoic teachings significantly enhanced resilience among medical students [31]. Resilience exhibits an inverse association with psychological distress [32]. Thus, resilience likely mediates the improvement in psychological distress. Moreover, these findings align with MacLellan et al., who demonstrated that Stoic training significantly improved rumination [20]. Stoic practices may mediate the observed reduction in psychological distress through their capacity to attenuate rumination. Various studies in recent years have demonstrated that Stoic approaches can improve mental health and enhance resilience across diverse populations [15, 16, 33].

Stoic philosophy, in contrast to prevailing perfectionism that pursues flawlessness in appearance or performance, emphasizes inner, moral, and intellectual excellence. This philosophical tradition maintains that individuals should cultivate virtue in their inner realm (beliefs, desires, and emotions), as external outcomes lie beyond their control. The Stoics advocate living according to cardinal virtues—rationality, justice, courage, and moderation—considering true perfectionism to consist in embodying these qualities [34].

From the Stoic perspective, many life circumstances—such as others' opinions and judgments, or genetic and chronic conditions like end‐stage renal disease—fall outside our sphere of control; in other words, some things in the world are up to us while others are not. Consequently, striving for perfection in unavoidable domains proves futile [35].

Stoicism posits that error is intrinsic to human nature, as humans are inherently evolving beings. Making mistakes does not reflect an existential flaw; rather, trial and error—and the courage to confront challenges—serve as opportunities for growth. True perfection manifests not in avoiding difficulties for fear of failure, but in taking responsibility for one's errors and correcting course.

The Stoics emphasize focusing on the present, rejecting regret over the past or anxiety about the future. An individual who lives mindfully and aligns their actions with inner values achieves a form of perfection. Perfection and problem‐solving strength thus entail conscious living, continuous self‐reflection, and striving for improvement, free from obsessive fixation or fear of adversity [36]. These recommendations and strategies appear to mitigate psychological distress in the face of life's adversities by fostering resilience, a capacity that enables individuals to navigate challenges with greater equilibrium [37].

In the present study, self‐efficacy did not show significant improvement. Simultaneously, some recent studies have indicated that certain educational interventions, such as Stoicism‐based approaches, have sometimes been ineffective in improving psychological indicators in hemodialysis patients [38, 39].

This finding contrasts with the results of MacLellan et al., who demonstrated that Stoic teachings enhanced self‐efficacy in individuals with high levels of worry [20]. Similarly, Brown et al. found that certain Stoic practices—particularly negative visualization—significantly boosted medical students' self‐efficacy [31]. Multiple studies have demonstrated that spirituality‐based interventions—particularly those resembling Stoic principles—can enhance self‐efficacy in hemodialysis patients [40, 41]. Given that hemodialysis patients often experience severe depression and fatigue, their receptiveness to training appears to be diminished [22]. Furthermore, had the number of training sessions for patients been increased, the between‐group differences would likely have reached statistical significance. This discrepancy may also stem from differing socioeconomic conditions, as Stoicism is likewise contingent upon socioeconomic and cultural circumstances [42, 43]. Also, religious beliefs are associated with higher levels of self‐efficacy. Given that the study was conducted within a religious culture, the level of self‐efficacy was likely high from the outset, and the interventions proved insufficient to bring about further improvement [41, 44, 45].

Stoic philosophers maintain that nothing in this world truly belongs to us—neither possessions, nor relationships, nor health—but rather represents a divine trust. They argue that reclaiming this trust constitutes a natural phase of life's cycle. Consequently, they advocate for constant mental preparation for the loss of blessings (through negative visualization), which reduces psychological attachment and emotion‐driven responses [46]. The Stoics emphasize a fundamental distinction between what lies within our control and what lies beyond it (The Trichotomy of Control Doctrine). Losses—whether of status, health, interpersonal relationships, or other external circumstances—typically fall outside our sphere of control. True tranquility emerges when we focus our efforts exclusively on domains we can influence [47].

These recommendations and strategies appear to enhance appropriate coping styles, thereby improving self‐efficacy when facing life's challenges and adversities. However, some narrative studies have criticized Stoic philosophy, arguing that promoting excessive tolerance for suffering discourages individuals from seeking psychological help. This reluctance to pursue professional assistance may reduce help‐seeking behavior and ultimately compromise mental well‐being [48, 49]. This stands in contrast to the Stoic philosophy, which urges individuals to endure, tolerate, and cultivate positive, adaptive responses only toward unavoidable and unchangeable suffering. From the Stoic perspective, one must take active measures to alleviate suffering that is controllable and mutable [50].

Stoic philosophy teaches individuals to confront life's challenges and suffering proactively—not through avoidance or escape, but through acceptance and even gratitude, embracing them as divine providence or destiny. At the same time, it encourages active resolution of controllable problems, as it is precisely in such circumstances that human ingenuity, courage, perseverance, resilience, and strength are revealed [47]. Iran presents a distinct sociocultural context compared to the environments where Stoicism originated and where it is mostly studied. Consequently, our research is the first to be conducted in this specific context and population.

### 4.1 Limitations

In the present study, the post‐test questionnaire was completed after a delay of only 1 month; further follow‐up is required. Future studies should administer post‐test questionnaires immediately after the intervention and include longer‐term follow‐up assessments.

Given that the study was conducted at a single center and involved non‐random sampling, it is advisable to exercise caution when generalizing the results. The participants were not blind; thus, awareness regarding the intervention might have influenced the effects. Furthermore, it is noteworthy that participants' feedback regarding the acceptability of Stoicism was not collected.

## 5 Conclusion

The findings suggest that instruction in Stoic principles may effectively reduce psychological distress. This study employed a dual‐modality approach, utilizing digital media (instructional videos and podcasts) and in‐person sessions for discussion and clarification. However, the results indicate that more extensive training, sustained practice, and additional scenario‐based exercises would be required to significantly improve self‐efficacy—a consideration researchers should address in future studies.

## Author Contributions

**Amirhossein Boostani:** conceptualization, investigation, writing – original draft, writing – review and editing, methodology, resources, project administration, visualization, supervision. **Amirmohamad Momeni:** project administration, conceptualization, investigation, writing – review and editing, methodology, software, data curation, formal analysis. **Mohammad‐Ali Cheraghi:** writing – review and editing, methodology, supervision, validation. **Fatemeh Salmani:** methodology, validation, software, formal analysis, data curation, supervision, writing – review and editing. **Mozhgan Moshtagh:** conceptualization, investigation, writing – original draft, writing – review and editing, methodology, project administration, formal analysis, resources, supervision, visualization.

## Funding

The authors have nothing to report.

## Consent

The authors have nothing to report.

## Conflicts of Interest

The authors declare no conflicts of interest.

## Transparency Statement

The lead author, Amirhossein Boostani, affirms that this manuscript is an honest, accurate, and transparent account of the study being reported; that no important aspects of the study have been omitted; and that any discrepancies from the study as planned (and, if relevant, registered) have been explained.

## Supporting information


Supporting File

## Data Availability

The data that support the findings of this study are available on request from the corresponding author. The data are not publicly available due to privacy or ethical restrictions.
