# Supplementary material for: The Effectiveness of a Stoicism‐Based Educational Package on Psychological Distress and Self‐Efficacy in Patients Under Hemodialysis: A Randomized Controlled Trial
Source: Health Sci Rep. 2026 Oct 5;9(10):e73336. doi: 10.1002/hsr2.73336 (PMC13635813; doi:10.1002/hsr2.73336)
Supplement: Supplementary file 1 [file HSR2-9-e73336-s001.docx]

| **Teachings Outline** | |
| --- | --- |
| **Sessions** | **Topics** |
| **-** | Introducing the intervention |
| 1 | Trichotomy of Control: Within the framework of this tenet, the dialysis patient was instructed to partition life circumstances into three tiers—uncontrollable, controllable, and partially controllable—and to practice adaptive acceptance of the former, given that preoccupation or struggle with such factors yields no constructive outcome. End-stage kidney disease was identified as a prototypical instance of an uncontrollable condition. |
| 2 | Premeditation of adversity: Within the framework of this tenet, the dialysis patient was taught to always be grateful for blessings and to recognize that the situation could have been much worse, so he/she should remain thankful. |
| 3 | Altruistic action: Within the framework of this insight, the patients were taught that if they reflect upon the blessings, they will realize that many of these blessings and facilities have been provided to them without any particular effort or hardship on their part. How wonderful it is, then, that since God has bestowed these blessings upon them freely, they too should share these blessings with others and give freely. |
| 4 | Contemplating determinism: Within the framework of this doctrine, the subjects were taught that existence is oriented toward the good. Therefore, they must accept the sufferings and hardships that cannot be changed and trust in divine destiny. |
| 5 | Social duty/ temperance: Within the framework of this teaching, the subject was instructed to practice moderation and balance in life affairs, to avoid excess and deficiency, and to actively participate in society while observing health guidelines. |
| 6 | Postponement of responses: Within the framework of this idea, the dialysis patient was taught not to make decisions when feeling emotional, and to postpone any decision until the emotions have subsided. |
| 7 | Separating Judgements from Events (Mindfulness): Within the framework of this doctrine, the subject was taught that human suffering is largely caused by internal factors—namely, one's desires, emotions, and beliefs. Therefore, one must constantly reflect upon one's inner state and separate external reality from subjective interpretation. |
| 8 | A summary of the previous topics (group discussion) |
